# An Autonomous City-Wide Light Pollution Measurement Network System Using LoRa Wireless Communication

**DOI:** 10.3390/s23115084

**Published:** 2023-05-26

**Authors:** Krystian Erwinski, Dominika Karpinska, Mieczyslaw Kunz, Marcin Paprocki, Jaroslaw Czokow

**Affiliations:** 1 Department of Automatics and Measurement Systems, Institute of Engineering and Technology, Faculty of Physics Astronomy and Informatics, Nicolaus Copernicus University, Grudziadzka 5, 87-100 Torun, Poland; 2 Department of Geomatics and Cartography, Faculty of Earth Sciences and Spatial Management, Nicolaus Copernicus University, Lwowska 1, 87-100 Torun, Poland; karpinskadominika@doktorant.umk.pl (D.K.);; 3 SPE Labs, Wloclawska 167, 87-100 Torun, Poland

**Keywords:** light pollution, LoRa, Torun metropolitan area, wireless measurement system

## Abstract

Light pollution is an ongoing problem for city populations. Large numbers of light sources at night negatively affect humans’ day–night cycle. It is important to measure the amount of light pollution in order to effectively ascertain the amount of light pollution in the city area and effectively reduce it where possible and necessary. In order to perform this task, a prototype wireless sensor network for automated, long-term measurement of light pollution was developed for the Torun (Poland) city area. The sensors use LoRa wireless technology to collect sensor data from an urban area by way of networked gateways. The article investigates the sensor module architecture and design challenges as well as network architecture. Example results of light pollution measurements are presented, which were obtained from the prototype network.

## 1. Introduction

Light pollution is a progressive degradation of the surrounding natural environment, which is defined as the excessive emission of artificial light into the lower atmosphere over an extended period of time. This phenomenon is currently global, and in recent decades, along with the development of industry and the progressing urbanization process, it systematically increases the spatial range of its impact. The glow of light extending over a city can easily be seen from a distance of up to several tens of kilometers [1,2,3]. According to research, over 99% of Europe’s population and 80% of the world’s population live in areas polluted by artificial light [1]. The interest in studying this phenomenon is systematically growing, and the group of scientists around the world who are involved in this research is expanding every year [4,5,6,7]. The growing interest in the issue is evidenced by the growing number of published scientific articles and conference presentations in recent years [8,9]. The phenomenon of artificial light pollution of the night sky has negative consequences for the environment. Excessive light emission leads to disturbances in the behavior of plants and animals and also significantly impairs human health, quality of life and everyday functioning [10,11]. The negative effect of this phenomenon is the presence of lighting in areas that should not be exposed to it, dazzling random people—both pedestrians and drivers—which can lead to dangerous situations, events and behaviors. In the energy crisis that has been progressing since the beginning of 2022, ill-considered, improperly designed outdoor lighting causes excessive electricity consumption, which leads to additional economic costs for local governments at all levels and individuals. For this reason, the development of measuring devices and the creation of a monitoring network seems very expedient and justified. Industry 4.0 creates new opportunities in this regard. Studies on the above-mentioned phenomenon in urbanized areas also show that light pollution increases further during the occurrence of unfavorable weather conditions, such as fog or cloud cover, and the increased presence of anthropogenic dust in the troposphere [12,13,14]. This type of comprehensive research is still not very widespread, but the demand of city authorities and agglomeration managers for a multi-threaded understanding and knowledge of the essential components of the formation of light smog is steadily increasing. This counteracts this process and, consequently, prevents its effects. Measuring, analyzing and interpreting the phenomenon of pollution of the night sky by artificial light is a very complex issue that requires multifaceted research and contributory work carried out in collaboration between people representing different scientific disciplines.

The phenomenon of light pollution in the night sky can be measured using various research methods. They can be divided according to specific criteria, e.g., according to the tools used, into instrumental methods and observational methods, or according to the complexity of the measurement process, into methods that can only be used by a qualified operator or a non-professional [2,5,7,15]. However, the most frequently used measurement methods in practice are registrations with the use of photometers and digital cameras, mainly wide-angle lenses or the “fish-eye” type, and their subsequent processing using specialized software [2,6]. Factory-made photometers can be both handheld, portable and stationary. Since 2020, an interdisciplinary team of scientists from the Nicolaus Copernicus University in Toruń has started work on designing, testing and implementing a proprietary automatic measuring device in the city, which is part of the light pollution monitoring system [16]. Measurements made in this way make it possible to compare the intensity of the phenomenon recorded at different locations [5,6]. In some research centres, studies using satellite or UAV imagery or aerial photographs are also used [17]. The most commonly processed images are those taken by the Suomi NPP satellite with the VIIRS instrument, the DMSP satellite with the OLS instrument and the Luojia 1-01 satellites [18,19].

## 2. Light Sensors

**Figure 1:**
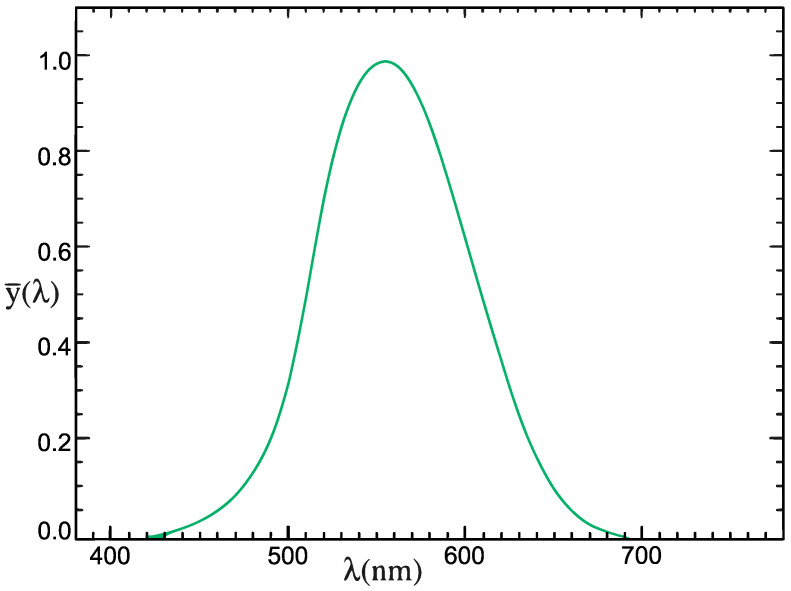
Photopic curve which models the sensitivity of the human eye to different wavelengths of the visible spectrum.

In order to measure and process the illuminance of ambient light, some kind of sensor is required. This sensor converts the value of illuminance in lux to a digital or analogue signal that can be processed by a microcontroller. The application presented in this paper requires the sensor to be low cost, accurate at low light conditions, have low power consumption and operate at voltages provided by batteries such as 3.3 V. The sensors also have to reflect the sensitivity of the human eye, which is modeled by the photopic curve [20] presented on Figure 1.

Electronic ambient light sensors can be based on several types of photosensitive elements such as light-dependent resistors (LDRs), which alter their resistance depending on the intensity of light or photo voltaic cells that generate voltage depending on light intensity. Another type of detector is the photodiode. The photodiode is a reverse-biased P-N junction which generates a small reverse leakage current proportional to the intensity of light. Because the leakage current is very small, the photodiode usually requires an amplifier to achieve useful signal levels. Phototransistors are two P-N junctions that work similarly to photodiodes; however, they also amplify the current so an additional amplifier is not needed. Among the previously mentioned light sensing devices, the photodiode is most widely used as an ambient light sensor. This is due to its fast response, low dependence on temperature and mostly linear illuminance vs. current characteristics. Due to its wide adoption as a light-sensing device, many semiconductor manufacturers offer integrated ambient light sensor chips which contain photodiodes, amplifiers, control electronics and an interface to transfer the measured value to a measurement system (usually a microcontroller). These include analog voltage or current signals, frequency signals or a serial communication interface such as SPI, I2C or UART. The main drawback of semiconductor devices used for ambient light sensing is their high sensitivity to infrared radiation. To alleviate this problem, filters are used which eliminate most of the infrared radiation and enforce spectral characteristics close to the photopic curve.

**Figure 2:**
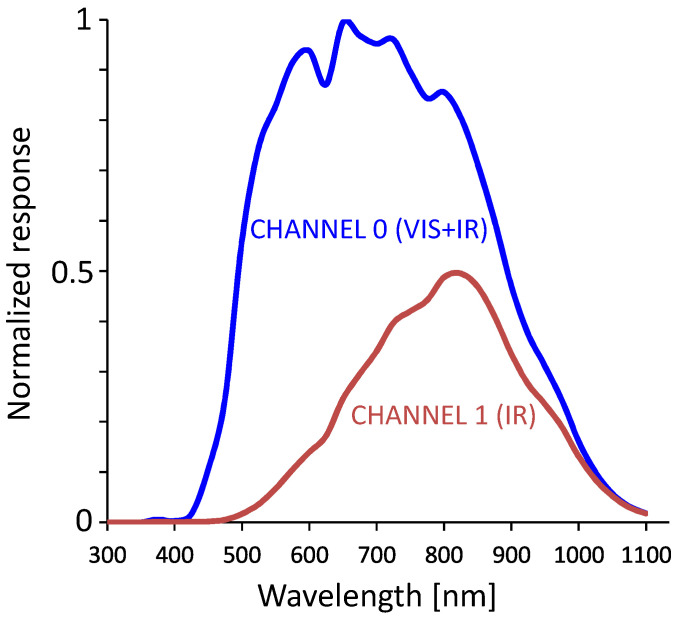
Spectral characteristics of both channels of the TSL2591 sensor.

In this work, an alternative solution was chosen in the form of a TSL2591-integrated ambient light sensor manufactured by AMS OSRAM. The sensor chip has two reverse-biased photodiodes. One is sensitive to infrared radiation and the other detects both infrared and visible spectrum. Both currents are measured using integrated amplifiers and converted to a numerical representation (16-bit integer) using integrated analog-to-digital converters (ADCs). The measured values can then be subtracted to eliminate the influence of infrared radiation and achieve the desired spectral characteristics. This allows us to eliminate the filter, which decreases the costs and complexity of the device [21]. The characteristics of the dual diode sensor are shown in Figure 2. The characteristics are inherent to the sensor and cannot be modified. The filter can still be used if the default characteristics are not satisfactory, but in most applications, they do not need to be adjusted. An alternative would be to use spectral sensors which can measure light intensities for several fragments of the visible light spectrum and infrared. The characteristics could then be adjusted to meet specific requirements by applying appropriate gains to each measured spectral intensity.

**Figure 3:**
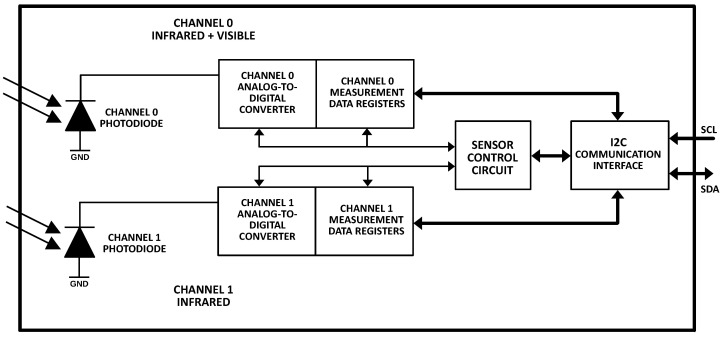
Block diagram of the TSL2591 sensor.

An exchange of data between the sensor and the microcontroller is achieved via I2C bus. Sending data via digital bus has the advantage of eliminating the influence of noise, which is a problem when using analogue signals. Furthermore, the bidirectional bus can be used to change parameters of the sensor, such as amplifier gain or integration time. The bus can also be used to set low and high limits for measured light, which trigger an interruption when violated. The sensor can also be put into low-power mode in order to conserve energy, which is useful in battery powered devices. A block diagram of the sensor is presented in Figure 3.

**Figure 4:**
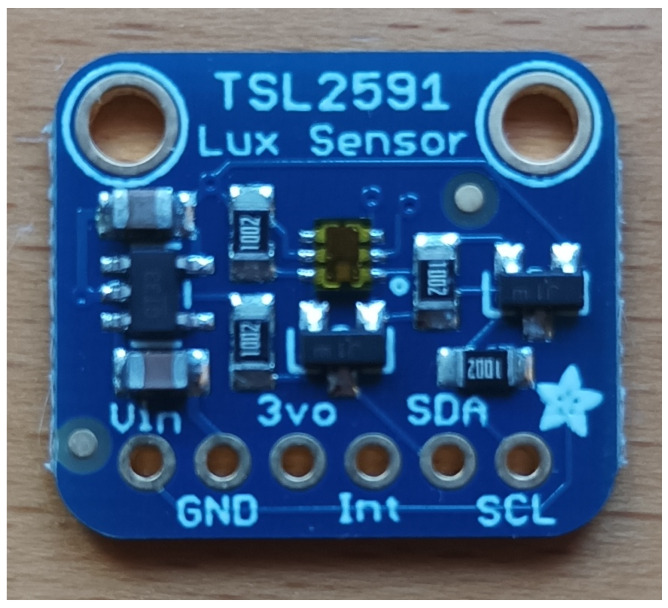
Adafruit module with the TSL2591 lux sensor.

The sensor can be purchased as a separate chip or as a small PCB module which contains all the necessary passive elements. A picture of an Adafruit module with the TSL2591 sensor is shown in Figure 4.

## 3. Remote Light Measuring Device

**Figure 5:**
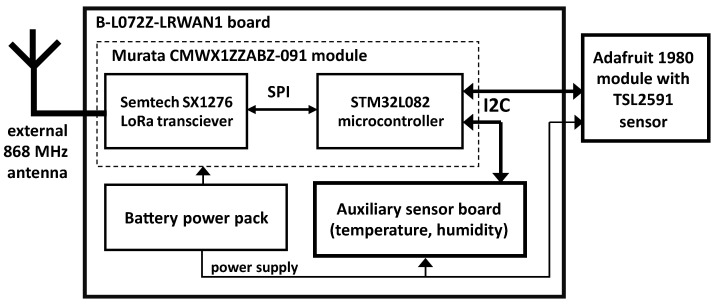
Block schematic of the LoRa light sensor.

The remote light measuring device, developed by the authors, is based on the B-L072Z-LRWAN1 board with the Murata CMWX1ZZABZ-091 processor. This processor integrates a low-power STM32L081 micro-controller and a Semtech SX1276 wireless transceiver compatible with the LoRaWAN standard. An Adafruit 1980 sensor board is used with the TSL2591 Lux sensor (Figure 3, left). The sensor communicates with the processor via I2C serial bus. The board is expanded with an auxiliary sensor board with temperature and humidity sensors to monitor the device during long-term outside operation for abnormal temperatures and leaks. The block schematic of the device is presented in Figure 5.

**Figure 6:**
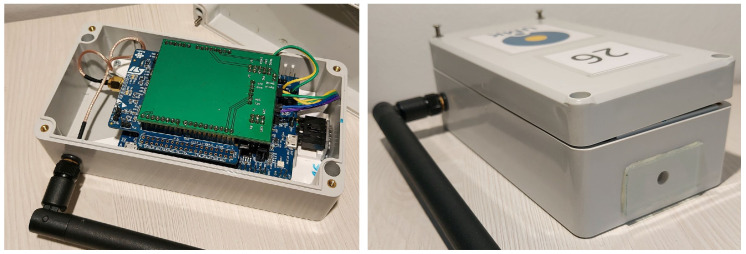
Pictures of the developed LoRa light measurement device (photo by Dominika Karpińska).

The boards are enclosed in a hermetically sealed plastic enclosure. The enclosure also contains a battery pack with three AAA batteries. An external antenna is attached to the enclosure and connected internally to the Murata chip. The sensor monitors the ambient light via a round hole covered by a thin glass window. The window is attached to the enclosure using a resistant marine silicone to protect the device against environmental conditions. The device should be placed on an elevated position so that the sensor window points upwards, toward the night sky. This allows measuring light pollution which is caused by excess light emitted to the atmosphere, where it is scattered and reflected back to the ground. Such placement also limits the influence of small local light sources placed near the device, which might significantly affect the reading while not contributing much to light pollution. Pictures of the developed LoRa light measurement device are shown in Figure 6.

The device is designed to operate for an extended period of time (at least one year) on battery power. Therefore, it was necessary to adapt the board hardware and properly configure the software to limit power consumption.

The board hardware was adapted by taking out unnecessary elements. Sensors on the additional sensor boards were soldered out, except for the temperature and humidity sensors. Some of these sensors were powered by power converters which were not necessary after the respective sensors were taken out. Furthermore, unnecessary pullup resistors for the I2C bus were also removed. The ST-Link programming interface which was also used to power the board via USB was also disabled and the board was powered directly from the battery holder.

Software modifications mainly included configuration of the microcontroller and LoRa module to revert to sleep mode when not used. The internal real-time clock (RTC) of the microcontroller was configured to wake up the hardware by interrupting at predefined times. The sensors were also configured to sleep when not used and were woken up when the processor initiated transmission. These hardware and software modifications allowed us to significantly reduce the current consumption.

Because the devices’ purpose was to measure artificial light pollution, the sky brightness measurements were conducted only at night. In order to properly discern the time of day, a real-time clock embedded in the processor was used. During the daytime, the processor and sensors are put in sleep mode to minimize energy consumption. The processor is woken up at 21:00. It then wakes up the LoRa transceiver and sensors. The measurements are received via I2C, processed and sent to the base station (gateway) using LoRa wireless network. After a successful transmission, the processor and all peripherals go to sleep for 15 min. After that period of time, the measurement and transmission cycle is repeated until 6:00. After that, the device goes into extended sleep until the next night.

At each measurement cycle data registers from both channels are read from the sensor via I2C. Because the registers are 16-bit, each one is read in two steps of one byte during one I2C transmission. After merging the bytes, two integer numbers are obtained with values ranging from 0 to 65,535. These are first converted to floating point numbers and used to compute brightness in Lux using the following formulas:

(1) $$CPL=\frac{IT*GAIN}{U}$$

(2) $$L_{1}=\frac{IR_{raw}-B\cdot FULL_{raw}}{CPL}$$

(3) $$L_{2}=\frac{\left(C\cdot IR_{raw}\right)-\left(D\cdot FULL_{raw}\right)}{CPL}$$

(4) $$L=\max\left(L_{1},L_{2}\right)$$

where: CPL—count per lux, $L_{1},L_{2}$—lux components from both channels, *L*—final value in lux with IR influence removed, IT—integration time, GAIN—sensor gain, $IR_{raw},FULL_{raw}$—raw integer values read from IR and FULL range channel registers, U,B,C,D—manufacturer-provided coefficients specific to the TSL2591 sensor, equal to 408, 1.64, 0.59, and 0.86, respectively. The equations and coefficients are based on documents provided by the manufacturer (AMS OSRAM) [22,23].

The user can set four different gains for the sensor (1, 25, 428, and 9876) and different sensor integration times between 100 and 600 ms. A higher integration time makes the sensor more sensitive in low-light conditions. Because this device is used for night sky brightness measurement, the highest gain of 9876 is used by default. The integration time is set to 400 ms by default. These values are adjusted dynamically so that if raw values are larger than 30,000 units, the gain and integration time is decreased. If the raw value is less than 200, the integration time is increased to 600 ms. The final floating point values are multiplied by 1,000,000 and converted back to integer form so that they can easily be sent over the LoRa network.

The data, in the form of integer lux values, are sent to the gateways immediately after being read from the sensors, after which the sensor goes to sleep until the next transmission cycle. Data from measurement devices are collected by their respective gateways. Data from each device are identified based on their unique LoRaWAN identifier and then forwarded to the central server with a timestamp using the internet (WiFi, Ethernet or LTE depending on the gateway location) via MQTT protocol. This is a private cloud server that can be accessed only by authorized personnel. The server hosts a node-red application that collects data from each gateway. The incoming data are stored in the server database and also CSV files for easy access and further analysis. There are also automated mechanisms to notify the user if any of the devices stop working, need a battery replacement (battery voltage measurement), have overheated (internal temperature measurement) or have had their enclosure compromised (internal humidity measurement). Additional data (temperature, humidity, and battery voltage are sent at the beginning and end of the measurement session. Apart from the automated signaling of abnormal states, the data analysis is performed offline by us on our local computers by downloading current data from the cloud. There is ongoing work to implement automated analytics in the cloud and develop a web-based front-end to visualize the collected data in a user-friendly manner. This will include a visualization available to the general public and a more detailed interface for dedicated users.

There are relatively very few examples of a device for autonomous long-term and long-range light pollution measurement in the literature. Compared to a similar device presented in the literature [24], our device has the benefit of being battery powered and optimized for low power consumption, so it does not need an external power supply or power source such as a photovoltaic cell. Furthermore, in the referenced work, an old sensor type was used, which is the same as in the Unihedron SQM photometer (commonly used in light pollution measurement). This sensor type requires a dedicated filter to eliminate the influence of infrared radiation on the light measurement, adding additional cost and complication to the device’s construction. Thanks to a more modern sensor and limitation of power consumption, our device is more cost effective than similar devices used for light pollution measurement.

The device developed by us has an additional advantage, because its housing allows for mounting both on poles, roofs or other structural elements of basically any type, as well as on drones. Thus, research was conducted at a vertical gradient. This has been described in [17] and is probably the first such application. In future, it will be a challenge to better understand this aspect of the phenomenon of light pollution.

Further improvements to the device will include development of a custom board, the addition of additional environmental sensors and alternative, more advanced light sensors, including biosensors [25,26,27].

## 4. LoRa Network

**Figure 7:**
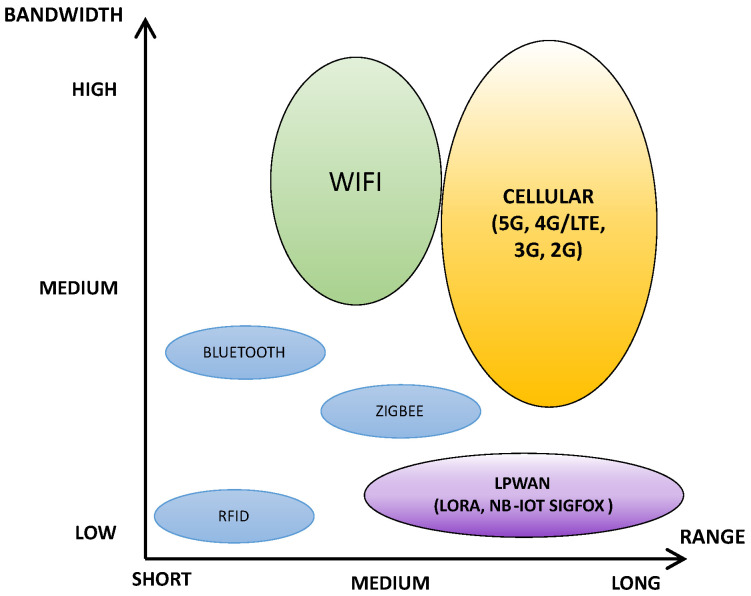
List the most popular communication protocols used in IoT—bandwidth and range comparison.

The current development of Internet of Things (IoT) devices [28] is associated with the 4.0 industrial revolution [29], which has been progressing for over ten years. IoT devices are often referred to as “smart” solutions in the context of their use—most often as sensors for monitoring certain physical quantities, e.g., for buildings, urban infrastructure or healthcare applications, etc. IoT devices should generally be characterized by low energy consumption and, thus, not too much computing power. IoT devices’ small size and low production costs are also desirable (mainly due to the mass production of this type of device). Another critical aspect of IoT devices is the type of communication interface involved. The most common type of communication is wireless communication. There are many variations in communication protocols used in IoT devices. The most popular protocols include Bluetooth [30], Zigbee [31] Wi-Fi [32], based on GSM networks (2G, 3G, 4G, 5G) [33], NFC [34] and others. An important aspect of such a communication protocol is the bandwidth-to-range ratio. Figure 7 lists the most popular communication protocols used in IoT devices, taking into account their bandwidth and range.

The Sigfox [35] and LoRaWAN [36] protocols, which are characterized by very long ranges (distance of kilometers) and low energy consumption during use, are particularly noteworthy. Such networks based on these communication protocols are called Low-Power Wide Area Networks (LPWANs). In the devices described in the article, the authors decided to use a network based on LoRaWAN (Long-Range Wide-Area Network), a long-range standard that uses a low data rate with low power consumption needs.

In LoRa networks (in Europe), the radio frequency used for data transmission is 433 MHz and 868 MHz. In the case of LoRaWAN, a frequency of 868 MHz band is used. LoRa networks use sharing mode—the band is split into several channels that can be used to transmit information. LoRa does not allow devices to transmit data continuously. However, in LoRa networks, devices using it are not immune to communication collisions. To solve this problem, spread spectrum modulation methods are used in LoRa. Chirp Spread Spectrum (CSS) [37] modulation is used.

The authors use the SX1276 chip from Semtech company [38] as a communication module with the LoRa network. The essential characteristics of this chip are summarized in Table 1.

**Figure 8:**
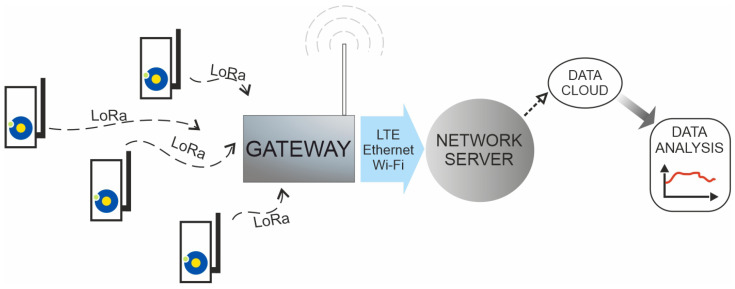
The architecture of the LoRaWAN network with the light intensity sensors.

The standard LoRaWAN network architecture consists of end devices, gateways, and servers. Data from light sensors (end-device) are sent to gateways via LoRaWAN communication. Data are then processed into a different protocol and sent (e.g., using Wi-Fi, GSM, or other technology) to the data aggregation server. The server also visualizes data from light-intensity sensors. The solution of the light measurement network based on the architecture of the LoRaWAN network is shown in Figure 8.

Due to duty-cycle restrictions in the European Union for the 868 MHz band, each of the end devices (light measurement sensor) should transmit no more than 1% of the time in which it works. In this case, sending a message takes about 3.5 s, which is less than 0.4% of one device work cycle.

To develop the architecture of a LoRa network, preliminary calculations regarding the data transmission time between devices and gateways should be made. This time depends on the Spreading Factor. The higher the Spreading Factor, the longer the transmission time in the LoRa network. Depending on the modulation used, this also affects the bandwidth. The transmission time of each symbol depends on the bandwidth used. This time is inversely proportional to the bandwidth. The higher the Spreading Factor, the lower the bit rate. The higher the bandwidth, the higher the bit rate.

In the presented application of light pollution measurement, long intervals between measurement are present, so the highest spread factor was used as there is a large amount of time available. This has the benefit of high robustness against interference and increased transmission range. No detailed calculations were necessary. In applications with shorter communication cycles, more attention should be paid to proper adjustment of the spread factor.

**Figure 9:**
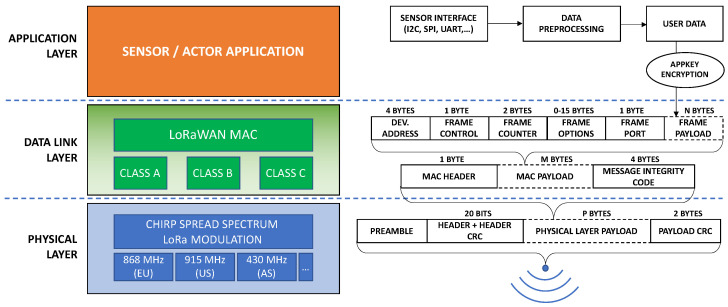
LoRaWAN protocol structure.

The LoRaWAN network is based on the OSI-type network model as standard. Therefore, the encapsulation of data sent in the LoRaWAN frame is also subject to changes (depending on the network layer) [39]. Figure 9 shows the content of the LoRaWAN frame, including individual data processed by subsequent layers of the LoRaWAN network.

## 5. Experimental Results

**Figure 10:**
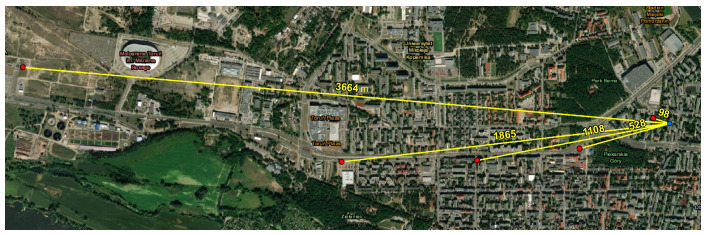
Locations of measuring devices (red) and LoRa gateway (blue) in Torun with distances.

**Figure 11:**
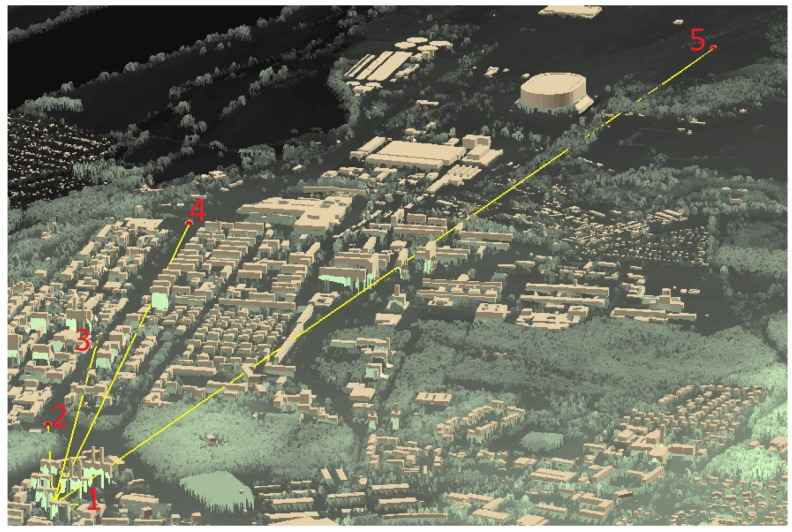
Locations of measuring devices marked on a numerical 3D model of the terrain with buildings.

Numerous experiments were carried out to verify correct operation of the developed LoRaWAN network and the constructed light measurement devices. One of them was a study determining the current consumption of a constructed device for measuring light intensity at night when sending a data frame at different distances from the communication gateway. The measurements were carried out in a built-up area, which corresponds to the operation of the equipment in the conditions of its intended use. The measurements were carried out by five devices placed in different locations, which were characterized by free access for the duration of the measurements. There were also no obstructions blocking the LoRaWAN signal in their close vicinity. Each of them was surrounded by free space, but it was impossible to avoid the presence of urban buildings to a greater or lesser extent. When analyzing the results, it is worth remembering that the quality of the LoRaWAN network signal is affected not only by the distance from the transmitter, but also by terrain obstacles. The measurement devices were located at a distance of 98, 528, 1108, 1865 and 3664 m from the communication gateway, respectively. A satellite image of Torun and a 3D terrain model with locations of the measuring devices are shown in Figure 10 and Figure 11, respectively.

**Figure 12:**
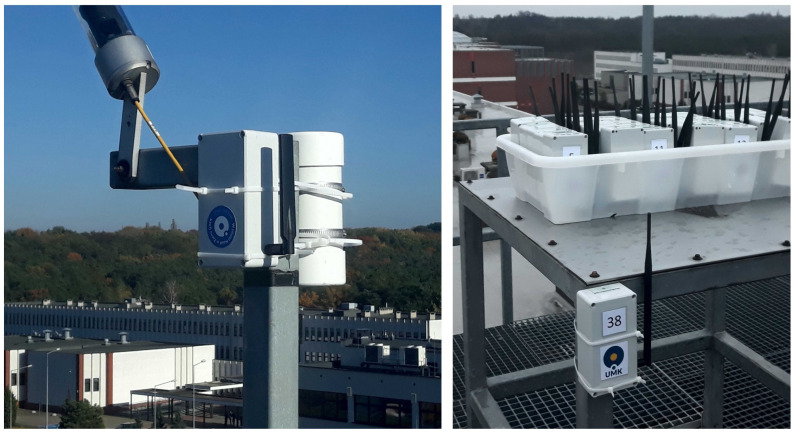
Pictures of the LoRa light measuring device deployed at outdoor locations (photo by Mieczysław Kunz).

Pictures of two devices deployed at different locations are shown in Figure 12.

**Figure 13:**
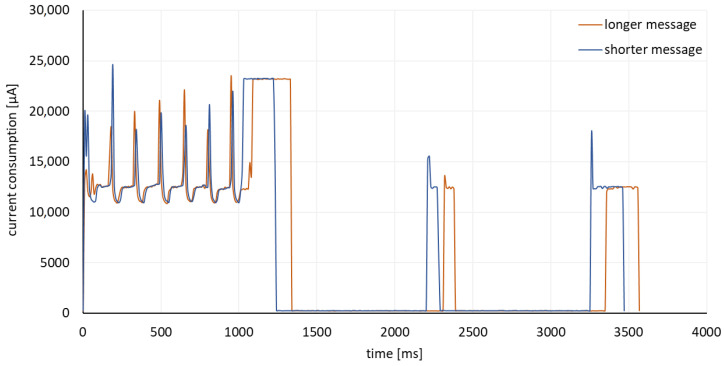
Sensor current consumption acquired using X-NUCLEO-LPM01A shield and STM32CubeMonitor-Power software while sending both long and short data frames.

Current consumption measurements were carried out using the X-NUCLEO-LPM01A shield, which is an extension of the STM32 Nucleo boards with power consumption measurement capabilities [40]. In order to read the measurements, it was necessary to use the STM32CubeMonitor-Power software, which enabled both reading parameters in real time and saving the collected data in the STPM file, storing data in the csv format. After collecting data in the same atmospheric conditions from all sites, it was possible to carry out further analysis of the results. From each of the measurements, characteristic waveforms were selected, showing the current consumption while the message was being sent. It is assumed that the constructed device sends two types of messages during one night measurement session, a short one with 32b, in which only the light intensity data are sent, and a long one with a length of 72b, which also sent data concerning the temperature, humidity and battery charge status. During one night, 36 messages are sent, including 2 long ones, monitoring weather conditions and device status. Figure 13 shows an example of power consumption when sending two types of messages.

The measurement and transmission interval was set to 15 min which is frequent enough to monitor the slowly changing night sky brightness. Each measurement and transmission cycle results in significantly more current consumption than sleep intervals between cycles. More frequent transmission intervals would result in more energy consumption globally, and therefore a shorter battery life. The interval was determined to achieve the longest possible interval which provides a satisfactory light value sampling rate.

After extracting several dozen samples showing the energy consumed while sending messages for each device, it was possible to average its value in each case. The obtained results were also averaged for the time when the device was in reduced power mode. For comparison, Table 2 shows the average maximum current consumption during device initialization, which takes place once after turning it on, and the average maximum current consumed while sending a data frame. Data were collected for each of the positions. Analyzing the data, it can be seen that the average during initialization remains similar for each device and does not depend on the distance between the device and the communication gate. However, we can see this dependence with the average power consumption during sending the message. As the distance increases, the average for a given position increases. The only exception is the site located 1108 m from the communication gateway. In its vicinity, there were dense multi-family buildings, which undoubtedly had an impact on the quality of measurements. Unfortunately, in urban conditions, it was not possible to choose a perfect location for the measurement device due to the presence of numerous terrain obstacles.

After averaging the data, it was possible to calculate the energy consumption of the device during a single initialization, sending a short and long message, as well as during the transition to a low-power state. With these data, the electricity consumption during one full day was calculated. This allowed us to determine the theoretical working time of the device on batteries with a capacity of 3000 mAh. The calculated data for each of the positions are presented in the Table 3.

**Figure 14:**
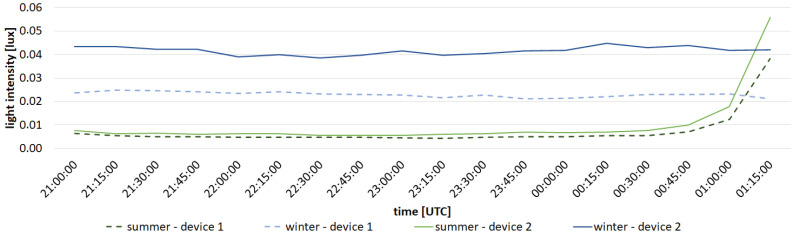
Comparison of measured light intensity for device 1 and 2 in summer and winter (average across all nights).

**Figure 15:**
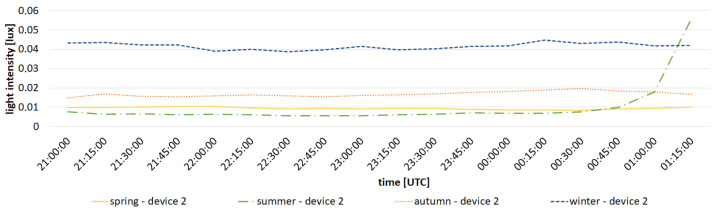
Comparison of measured light intensity for device 2 in all seasons (average across all nights).

Analyzing the data presented in the Table 3, we can see that the differences in energy consumption during one night measuring session between different devices are noticeable. Power consumption during night measurement session of device 4, which had the highest power consumption, is 13.5% higher than that of device 1, which has the lowest power consumption. The differences are mainly related to current consumption during transmission, which in turn is related to distance and any obstructions between the gateway and each device. However, the power consumption during the long sleep time of the device has the greatest impact on the available operational period. This sleep time is the same for each device. The total time taken to send a small amount of messages is very short compared to the length of the entire standby period, which is the cause of very similar estimated battery life in Table 3 The presented data prove that the distance and terrain obstacles only slightly affect the working time of the device. The highest and lowest results differ by less than 3 days, which is less than 0.6% of the calculated life. The above analysis proves how important it is to use technology that allows the use of reduced power consumption and allows sleep mode to be entered between measurements. This allows them to be placed in a location with limited access and uninterrupted archiving of data for a long time. Measurement devices, designed and built by the authors, that transmit data via LoRa technology have been operating in Toruń since April 2020. Because of this, it was possible to acquire preliminary seasonal results at several locations. Figure 14 presents a comparison of results for devices 1 and 2 in summer and winter, while Figure 15 presents results for device 2 for all seasons. Measurements are shown between 09:00 pm and 01:15 am due to the presence of a shorter astronomical night in the summer.By analyzing the presented data, one can notice the seasonal variability in the brightness of the night sky. Winter nights are definitely the brightest, while summer nights are darkest. The presented results are in agreement with the results obtained by other research teams monitoring the brightness of the night sky [41,42,43], as well as with the previous instrumental studies conducted in Toruń [4,44].

It is also worth noting that there is a noticeable difference between results obtained from different devices. The sky is brighter at the location of device 1 in winter compared to the location of device 2. In summer, the results for both devices are very similar; however, a slightly larger value for device 2 can still be noticed. This can be attributed to a different location of both devices with different lighting conditions, which also proves the usefulness of the developed system. The difference between seasons can be attributed to differences in the number of cloudy nights and the amount of air pollution, which influences light reflection from the atmosphere.

The collected data prove the relevance of the selected technologies and components for the construction of devices and the operational readiness of the constructed device in all measurement conditions. Measurements were taken both during the frost of several degrees and during the hottest days of the year. The communication of the device with the communication gateway was also uninterrupted under all conditions. Data archiving proceeded without any major disruptions. The conducted measurement sessions prove the readiness to work of both self-constructed devices and the entire prepared measurement infrastructure.

## 6. Conclusions

In this paper, an autonomous distributed light measurement system was presented for long-term monitoring of light pollution in an urban environment. The system consists mainly of autonomous measurement devices equipped with LoRa wireless communication, which allows long-range communication in various environments. Compared to similar devices presented in the literature, the device and data acquisition system presented in this paper have the advantage of autonomous long-term operation under battery power with wireless communication to a central network server. The paper presents the developed wireless sensor module and system architecture, as well as design challenges that had to be overcome to make the system operational. Experimental results were presented which prove that the devices are capable of operating in the LoRa network-based system for extended periods of time without maintenance due to low power consumption and an appropriate transmission schedule. Furthermore, long-term measurements of night sky light pollution were presented. These were gathered by several devices in the Torun urban area. The developed system and devices can be used, among other applications, to optimize urban lighting in different seasons in order to lower city-wide energy consumption. The system will be further developed to provide a web-based front-end available for general use and administrative purposes. Currently, 40 devices are being deployed across Torun, which should provide a much more detailed map of urban light pollution in that area. A new version of the device with a dedicated integrated board is currently being developed.

## Figures and Tables

**Table 1:** Main features of the SX1276 LoRa Transceiver.

Main Features
168 dB maximum link budget
+14 dBm high-efficiency PA
Low RX current of 9.9 mA, 200 nA register retention
Programmable bit rate up to 300 kbps
High sensitivity: down to −148 dBm
Packet engine up to 256 bytes with CRC

**Table 2:** Comparison of average maximum current consumption values during initialization and frame transmission for each measurement device.

Measuring Device	Distance [m]	Average Maximum Current Consumption during Initialization [mA]	Average Maximum Current Consumption during Transmission [mA]
Device 1	98	35.100	19.075
Device 2	528	35.781	19.349
Device 3	1108	34.881	25.871
Device 4	1865	34.698	21.043
Device 5	3664	35.742	35.206

**Table 3:** Comparison of average energy consumption of each device.

Distance [m]	Initialization [MAh]	Sending Long Message [mAh]	Sending Short Message [mAh]	Night Measurement Session [mAh/day]	Daytime Sleep [mAh/day]	3000 mAh Battery Life [Days]
98	0.0233	0.0068	0.0058	0.2122	5.942	507.92
528	0.0233	0.0068	0.0059	0.2137	5.942	507.79
1108	0.0233	0.0070	0.0063	0.2266	5.942	506.69
1865	0.0233	0.0068	0.0060	0.2179	5.942	507.43
3664	0.0233	0.0074	0.0068	0.2454	5.942	505.08

## Data Availability

Not applicable.
